# Massive Gastric Hemorrhage due to Dieulafoy's Lesion in a Preterm Neonate: A Case Report and Literature Review of the Lesion in Neonates

**DOI:** 10.1155/2015/937839

**Published:** 2015-06-08

**Authors:** Christos Salakos, Panayiota Kafritsa, Yvelise de Verney, Ariadni Sageorgi, Nick Zavras

**Affiliations:** ^1^Department of Pediatric Surgery, ATTIKON University Hospital, 1 Rimini Street, Haidari, 12462 Athens, Greece; ^2^Department of Pediatric Surgery, “IASO” Maternity and Children's Hospital, 37-39 Kifisias Street, Marousi, 15123 Athens, Greece; ^3^Department of Gastroenterology, “IASO” Maternity and Children's Hospital, 37-39 Kifisias Street, Marousi, 15123 Athens, Greece; ^4^Neonatal Intensive Care Unit, “IASO” Maternity and Children's Hospital, 37-39 Kifisias Street, Marousi, 15123 Athens, Greece

## Abstract

Dieulafoy's lesion is an extremely rare cause of upper gastrointestinal bleeding in the neonatal age group. Till now, only 6 cases of Dieulafoy's lesion in neonatal period have been reported in the international literature. Herein, we report an extremely rare case of Dieulafoy's lesion in a preterm neonate.

## 1. Introduction

Dieulafoy's lesion (DL) is a distinct entity characterized by the presence of a large artery located under the muscularis mucosa and usually protruding into the gastric lumen [1]. The lesion accounts for 0.3% to 6.7% of the upper gastrointestinal (GI) tract bleeding cases in adults [2]. However, its exact prevalence in the pediatric population is unknown as most published studies concern case reports. In a recent review of the English language literature, the authors identified 28 pediatric cases with DL, among whom there were two full-term neonates and one preterm. All these neonates manifested the disease on the 1st, 3rd, and 4th postnatal day, respectively [3–5].

Herein, we describe a preterm neonate with DL. A brief review on neonatal cases is discussed.

## 2. Case Report

**Figure 1:**
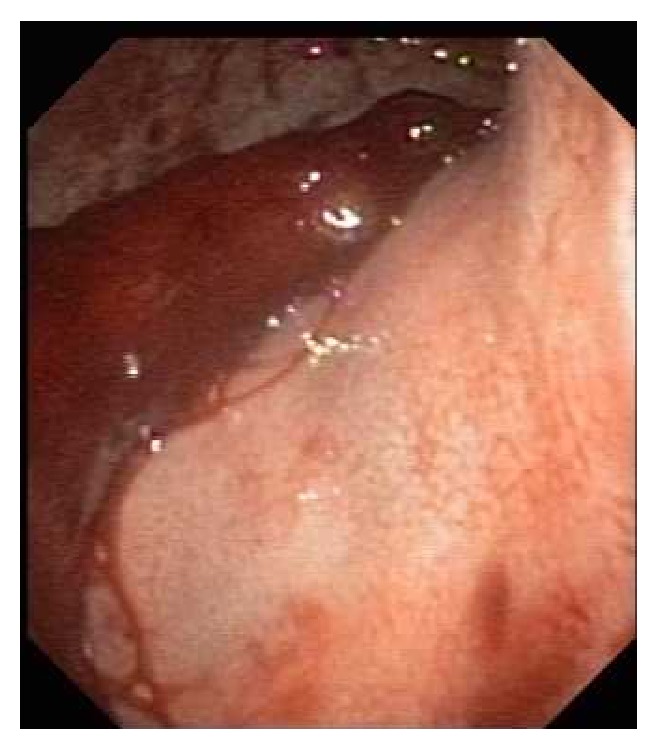
A big blood clot adherent to the gastroesophageal junction was seen in the 1st endoscopy. The clot could not be reached by endoscopy.

**Figure 2:**
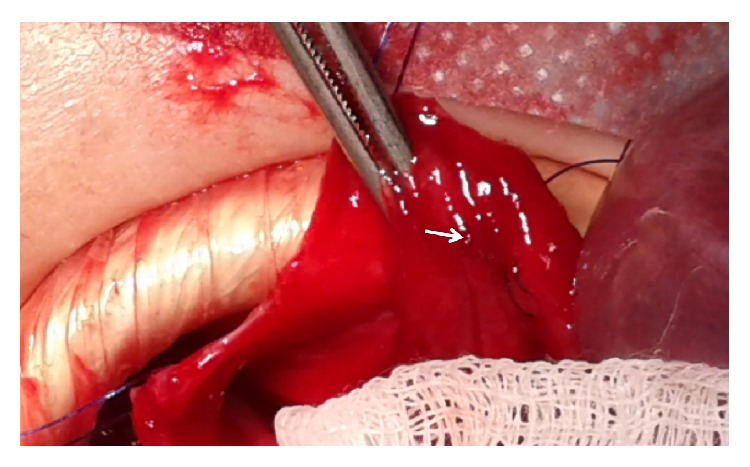
A spurting arterial vessel was seen after gastrotomy (white arrow).

A preterm male neonate was born as twin B after an IVF pregnancy to a 36-year-old gravida 1, para 2 mother at 26^+1^-week gestation, due to idiopathic preterm labor. His birth weight was 1010 g (90th percentile), and his length and head circumference were 34 cm (50th percentile) and 25.4 cm (90th percentile), respectively. Apgar scores were 4 at 1 minute and 6 at 5 minutes. The neonate was intubated to increase respiratory efforts and he was transferred to the neonatal intensive care unit (NICU). He remained on mechanical ventilation for 36 days. On day 65 of hospitalization (postconceptual age: 34^+5^ weeks, weight 2020 g), he presented with a massive oral hematemesis and was transferred to the NICU yet again. On examination, he was pale and displayed mild abdominal distention. Initial laboratory examinations showed hemoglobin of 10 g/dL (13.5–19.5 g/dL); hematocrit 29.3% (40–64%); WBC 5,050 cells/*μ*L (10.000–26000 cells/*μ*L); and platelet count 190,000/*μ*L (150,000–400,000/*μ*L). The coagulation tests were normal. After a one-blood volume transfusion, esophagogastroduodenoscopy (EGD) was performed with an Olympus GIF-N180 neonatal endoscope, which identified the presence of a big blood clot in the fundus, adherent to the gastroesophageal junction without signs of active bleeding (Figure 1). However, despite the efforts, the blood clot could not be reached even in full retroflexion. A second endoscopy was performed the following day after a massive hematemesis and a further drop in the levels of hemoglobin and hematocrit (8.6 g/dL and 25.1%, resp.). However, the presence of a pool of blood prevented the endoscope from visualizing the source of bleeding. Again, the site of bleeding could not be approximated. An emergency laparotomy with gastrotomy was carried out which revealed a spurting arterial vessel that was ligated at a distance of less than 3 cm from the gastroesophageal junction (Figure 2). The infant had an uneventful recovery. No recurrence of bleeding was noted during 8 months of follow-up.

## 3. Discussion

DL lesion is extremely rare in the neonatal age group. Relevant articles in the international literature, dating from the first case reported in 1968 [6] to present, were retrieved from PubMed, SCOPUS, and Medline using the key words Dieulafoy's lesion, caliber persistent artery, neonates, and children. We found only three cases in the English literature [3–5] and another three in the Asian literature [7, 8]. All but one neonate were full-term, with a male/female ratio 5 : 1 (Table 1). To our knowledge, this case is the second to be described in a preterm male neonate.

Although DL was first reported by Gallard in 1884 [9], it carries the name of Dieulafoy who reported the lesion in three patients with upper gastrointestinal bleeding [10]. He called it “exulceratio simplex,” characterised by an oval or elliptical shaped acute ulcerative process with dimensions of 2–5 mm. Regardless of the age of the patient, the lesion is usually located in the upper stomach 6 cm below the esophagogastric mucosa [11], but it can also be found anywhere within the entire GI tract from the esophagus to the rectum, or at sites outside the GI tract, such as the bronchi [12]. In the reviewed cases, including our own, DL was located in the stomach in all neonates (Table 1).

The clinical presentation more commonly includes a painless and massive upper GI hemorrhage, possibly recurrent [12]. Hemodynamic instability may be involved [12]. The pathogenesis of DL is not clearly understood. Among various causes, the hypothesis of a congenital origin seems to be the most acceptable [13]. According to this theory, the bleeding artery maintains its initial diameter as it enters the gastric wall rather than decreasing [13]. This abnormality renders the vessel prone to massive bleeding. The congenital anomaly is supported by reported cases of neonates.

Endoscopy is the first method of diagnosis in DL, and the diagnostic criteria are well established [14]. In cases of gastric DL in both the adult and pediatric population, the success rate ranges between 70% and 80% [11, 14], though repeated endoscopies may be required to establish the diagnosis [14]. Similarly, in the cases listed here (Table 1), the success rate of endoscopic diagnosis was as high as 85.7%. However, in our case, two endoscopic investigations failed to localize the source of bleeding due to the presence of a clot in the first endoscopy and active bleeding in the second; thus, a gastrotomy was performed to identify the source of bleeding.

So far, there is not a general agreement on the management of DL [14]; hence, the choice of treatment relies on the location of the DL and suitable skills. It is worth saying that advancements in endoscopic procedures, even in neonates, have limited the use of surgical intervention. A success rate reaching 98% has been reported with various endoscopic modalities, including the hemoclip, injection of sclerosants, thermocoagulation, or band ligation [3, 15]. In neonatal case series (Table 1), successful hemostasis was achieved with a hemoclip in three patients, injection of epinephrine in two patients, and thermal coagulation in one patient. Recently, surgical intervention was reserved only for cases deemed unmanageable with endoscopic procedures [3]. In our case, the site of the DL was very close to the gastroesophageal junction and was covered with blood. Consequently, an open laparotomy was decided. No recurrence was noted in our listed cases, and death was reported in just one patient [5] due to coexistent lesions.

The present case illustrates a very rare case of DL in a preterm neonate. Although endoscopy is considered the first diagnostic tool of intervention, in the paediatric population, the management of DL may be very difficult. The lack of fine endoscope and the tiny size of the stomach pose a challenge for endoscopic procedures. Notably, in the cases of recurrent massive bleeding of upper GI, an open approach should be promptly carried out to determine and treat DL.

## Figures and Tables

**Table 1:** Published cases of Dieulafoy's lesion in the neonatal age group.

Authors	GA	Sex	Age	Site	Diagnosis	Treatment	Recurrence	Outcome
Lee et al. [4]	Full-term	M	3 d	Stomach	Endoscopy	Hemostatic clip	No	Successful

Koo et al. [7]	Full-term	M	1 d	Stomach	Endoscopy	Endoscopic epinephrine injection	No	Successful

Koo et al. [7]	Full-term	F	1 d	Stomach	Endoscopy	Endoscopic epinephrine injection	No	Successful

Lee et al. [8]	Full-term	M	1 d	Stomach	Endoscopy	Hemostatic clip	No	Successful

Polonkai et al. [5]	Late preterm	M	5 d	Stomach	Endoscopy	Hemostatic clip	No	Death

Zavras et al. [3]	Full-term	M	1 d	Stomach	Endoscopy	Thermocoagulation	No	Successful

Present case	Preterm	M	65 d	Stomach	Laparotomy	Laparotomy	No	Successful
